# Aberrant Striatal Value Representation in Huntington's Disease Gene Carriers 25 Years Before Onset

**DOI:** 10.1016/j.bpsc.2020.12.015

**Published:** 2021-09

**Authors:** Akshay Nair, Eileanoir B. Johnson, Sarah Gregory, Katherine Osborne-Crowley, Paul Zeun, Rachael I. Scahill, Jessica Lowe, Marina Papoutsi, Stefano Palminteri, Robb B. Rutledge, Geraint Rees, Sarah J. Tabrizi

**Affiliations:** a Huntington’s Disease Centre, University College London Queen Square Institute of Neurology, University College London, London, United Kingdom; b Max Planck University College London Centre for Computational Psychiatry and Ageing Research, University College London Queen Square Institute of Neurology, University College London, London, United Kingdom; c University College London Institute of Cognitive Neuroscience, University College London Queen Square Institute of Neurology, University College London, London, United Kingdom; d Wellcome Centre for Human Neuroimaging, University College London Queen Square Institute of Neurology, University College London, London, United Kingdom; e Laboratoire de Neurosciences Cognitives et Computationnelles, Institut National de la Santé et de la Recherche Médicale, Paris, France; f Département d’Etudes Cognitives, Ecole Normale Supérieure, Paris, France; g Université de Paris Sciences et Lettres, Paris, France

**Keywords:** Computational psychiatry, Huntington's, Learning, Neuroimaging, Striatum, Value

## Abstract

**Background:**

In this study, we asked whether differences in striatal activity during a reinforcement learning (RL) task with gain and loss domains could be one of the earliest functional imaging features associated with carrying the Huntington's disease (HD) gene. Based on previous work, we hypothesized that HD gene carriers would show either neural or behavioral asymmetry between gain and loss learning.

**Methods:**

We recruited 35 HD gene carriers, expected to demonstrate onset of motor symptoms in an average of 26 years, and 35 well-matched gene-negative control subjects. Participants were placed in a functional magnetic resonance imaging scanner, where they completed an RL task in which they were required to learn to choose between abstract stimuli with the aim of gaining rewards and avoiding losses. Task behavior was modeled using an RL model, and variables from this model were used to probe functional magnetic resonance imaging data.

**Results:**

In comparison with well-matched control subjects, gene carriers more than 25 years from motor onset showed exaggerated striatal responses to gain-predicting stimuli compared with loss-predicting stimuli (*p* = .002) in our RL task. Using computational analysis, we also found group differences in striatal representation of stimulus value (*p* = .0004). We found no group differences in behavior, cognitive scores, or caudate volumes.

**Conclusions:**

Behaviorally, gene carriers 9 years from predicted onset have been shown to learn better from gains than from losses. Our data suggest that a window exists in which HD-related functional neural changes are detectable long before associated behavioral change and 25 years before predicted motor onset. These represent the earliest functional imaging differences between HD gene carriers and control subjects.

SEE COMMENTARY ON PAGE 854

Huntington's disease (HD) is a neurodegenerative disorder associated with a complex psychiatric, cognitive, and motor phenotype (1,2). HD is caused by a CAG triplet repeat expansion in the huntingtin gene (3,4). The length of this expansion is proportional to the age of onset of motor signs, which typically starts between 40 and 50 years of age (5). Although unequivocal motor features of HD are first seen at this point, neural atrophy and cognitive deficits are present earlier, in the premanifest phase (6, 7, 8). Given that disease-related changes occur before clinical diagnosis and with disease-modifying drugs for HD currently under investigation, an open question remains—what are the earliest changes associated with carrying the HD gene (9,10)?

HD is characterized by a range of cognitive deficits, particularly in fronto-executive functions such as attention, processing speed, set-shifting, and emotion recognition (11, 12, 13). Underlying these cognitive deficits is neuronal atrophy or dysfunction secondary to mutant huntingtin (*mHTT*) expression. In particular, the medium spiny neurons within the striatum, especially those within the indirect pathway, are highly susceptible to the HD disease process (14, 15, 16). In recent years, it has also become clear that the striatum is a key node in learning to maximize rewards (17, 18, 19, 20, 21). In keeping with these findings, differences in reward processing tasks have been found in persons carrying the HD gene (HDGCs), both in the manifest disease and in the premanifest phase up to 10 years before onset (22, 23, 24, 25, 26).

Learning to maximize rewards requires symmetrical performance in both learning to gain rewards and learning to avoid losses. Although these two processes involve different brain regions, both have been shown to activate the striatum (27, 28, 29). This is perhaps expected, as many computational models place the striatum at the center of behavioral policy adaptation that must occur in both pursuing gains and avoiding losses (30, 31, 32). Intriguingly, Palminteri *et al.* (23) reported in a behavioral study that premanifest HDGCs approximately 9 years from motor onset showed an asymmetry between gain and loss learning. In their study, premanifest HDGCs completed a reinforcement learning (RL) task with both gain and loss domains. They found that premanifest HDGCs showed better learning from gains than from losses—a “reward bias.” Computational modeling in this behavioral study further suggested that this effect was mediated by noisier decision making in the loss frame (23). Given the early involvement of the striatal indirect pathway in HD and the proposed role this pathway plays in learning from losses, such an asymmetry between gain and loss learning may be predicted as an early change associated with carrying the HD gene (14,31,33, 34, 35, 36, 37).

On this basis, we asked whether a reward bias could be one of the earliest functional imaging features associated with carrying the HD gene. To address this question, we undertook a functional magnetic resonance imaging (fMRI) study in which young healthy HDGCs who were predicted, based on CAG length and age, to be 25 years from clinical diagnosis completed an RL task to gain rewards and avoid losses. We asked whether there was evidence of an asymmetry between gain and loss learning in the HDGCs, compared with well-matched control participants, either at a behavioral level or in the corresponding fMRI signal. We looked for such a reward bias in frontostriatal regions of interest (ROIs) at both cue and outcome presentation and used computational modeling to better understand our findings.

## Methods and Materials

### Subject Details

We recruited 35 HDGCs and 35 matched control subjects who did not carry the HD gene. All participants were between 18 and 40 years of age. HDGCs were required to show no diagnostic motor features of HD (diagnostic classification score <4), have a CAG repeat length ≥40, and have a disease burden score ≤240, indicating that these patients are estimated to be at least 20 years from onset of motor symptoms. Control participants were required to have no known family history of HD or to have been tested for HD. Exclusion criteria were substance abuse, the use of any medications to treat HD, unstable dose of antidepressants in the past 30 days, and significant medical or psychiatric disorder. Groups were matched for age, sex, highest education level, and handedness. Demographics for both groups can be found in Table 1. This study was a separate substudy within the Huntington’s Disease Young Adults Study and was approved by the Bloomsbury Research ethics committee (16/LO/1323) (38).

**Table 1:** Sample Demographics

	HD Gene Positive, *n* = 35	HD Gene Negative, *n* = 35	*p* Value
Age, Years	29.4 (5.7)	30.5 (5.2)	.41^a^
Sex, Female, *n*	19	20	.81^b^
Handedness, Right, *n*	30	32	.70^b^
NART	102.2 (6.9)	103.4 (8.3)	.52^a^
Depressive Score	32.2 (8.6)	34.6 (7.4)	.22^a^
Adjusted Caudate Volume, mL	7.27 (0.75)	7.42 (0.67)	.40^a^
UHDRS Motor Score, Median (Maximum)	0 (5)		
CAG Repeat Length	41.8 (1.2)		
Disease Burden Score	185.1 (33.5)		

Values are reported as mean (SD) unless otherwise stated.

Sample demographics for both groups show that the cohorts were well matched at recruitment for age, sex, and handedness. Groups were matched for intelligence, as measured by the NART, and for depressive symptoms, as measured on the Zung Depressive Scale. Groups were in the normal range for depressive symptoms (<50). Caudate volumes adjusted for total intracranial volume (see Methods and Materials) were also not significantly different between groups.

HD, Huntington's disease; NART, National Adult Reading Test; UHDRS, Unified Huntington's Disease Rating Scale.

a The *p* value was calculated via independent *t* test.

b The *p* value was calculated via χ^2^ test.

### Task Description

The task used in this study was identical in design to that described by Pessiglione *et al.* (29) and Palminteri *et al.* (23) to allow for meaningful comparison between results (see Figure S1). Participants completed an RL task in which the aim was to maximize rewards by learning to choose the best symbol from a pair of abstract symbols displayed on the screen. Participants saw 3 pairs of symbols, corresponding to the 3 conditions—gain, neutral, and loss. In each condition, one symbol was associated with an outcome with probability of 0.8 and the other symbol was associated with the same outcome with probability of only 0.2. In the gain condition, this outcome was winning a fictional £1, signified by an image of a £1 coin with a green surrounding halo. In the loss condition, this outcome was losing a fictional £1, signified by an image of a £1 coin with a red cross superimposed over it. In both conditions, the alternative outcome was to receive nothing, signified by the word “Nothing” appearing on-screen. In the neutral condition, the two outcomes were either an empty gray disc or the word “Nothing” signifying no reward. For details of fMRI task presentation, see Supplemental Methods.

Participants were shown each pair of stimuli 30 times, with a total of 90 choices per run. Participants were given instructions and carried out 1 run of the task outside of the scanner to familiarize themselves with the task. Participants completed 2 runs in the scanner, with each run lasting on average 12 to 14 minutes. In the scanner, instructions were repeated before the task began.

### Behavioral and Computational Analysis

Behavior from both runs was concatenated, and an RL model of behavior was fit to the subject data across the 2 runs. Percentage correct was determined by the number of times a subject chose the best symbol in the gain and loss pairs. The difference between the percentage correct in gains and losses was used to compute the reward bias term. Differences between groups were tested using either rank sum tests or independent *t* tests, where appropriate, after assessment of data distribution, with *z* and *t* statistics reported, respectively. In all tests, *n* = 35 in each group. All behavioral analyses were completed in MATLAB R2017a (The MathWorks, Inc., Natick, MA).

Behavior was modeled using a *Q*-learning model, combining a Rescorla–Wagner learning model with a softmax action selection mechanism. The value of the chosen option (*Q*) was updated for the next trial (*i* + 1) by updating its current estimated value with the prediction error (δ) multiplied by the learning rate (α) (39):

$$Q_{a\left(i+1\right)}=Q_{a\left(i\right)}+\text{α}*{\text{δ}}_{\left(i\right)}$$

The prediction error term was calculated as follows:

$$\delta_{\left(i\right)}={\text{reward}}_{\left(i\right)}-Q_{a\left(i\right)}$$

where reward_(*i*)_ represents the outcome following choice: +1 for gaining money, 0 for nothing, and −1 for losing money. Action selection was modeled using the softmax function, in which the probability of choosing an action is determined as follows:

$$P_{a}\left(t\right)=\frac{e^{Q_{a}\left(t\right)*\text{β}}}{\sum_{i=1}^{n}e^{Q_{i}\left(t\right)*\text{β}}}$$

Using this action selection rule, the probability that an action is chosen is based both on its relative value to the other option and on a computational term, choice temperature (β), which represents choice stochasticity. Based on model comparison, initial *Q* values for both symbols for gain domain choices were set at 0.5 and −0.5 for loss domain. This model provided a good fit for both gains and losses, with no difference of fits between groups, and it performed best in terms of model comparison across 6 competing models. For details on model fitting, see Supplemental Methods and Figure S2.

### MRI Acquisition

Alongside acquisition of a T1-weighted sequence and field maps, functional imaging data were collected using a standard 2-dimensional echo-planar imaging sequence on a 3T Siemens Prisma system (Erlangen, Germany). A sequence optimized to minimize drop-out in regions near the striatum, orbitofrontal cortex, and amygdala was used (40). For details on MRI acquisition and structural image processing, please see Supplemental Methods.

### Functional Imaging Preprocessing and Modeling

Standard echo-planar imaging preprocessing steps were taken; see Supplemental Methods. At the participant level, a general linear model (GLM) was constructed, with cue onsets and outcome onsets as regressors of interest. Cue onsets were subdivided by valence and whether the button was pressed (i.e., regressors for gain cues were Gain-Go and Gain-NoGo). A second GLM was built for each subject for the computational model-based fMRI analysis containing the cue and reward onsets. The model-derived *Q* values and reward prediction error (RPE) from the computational model above were then used as parametric modulators at the time of choice and outcome, respectively. As no group difference in parameter estimates was found (see Results), individual *Q*-value regressors and RPE regressors were estimated from each subject’s choice behavior using the mean learning rate parameters across the whole group (*n* = 70, α_gain_ = .122, α_loss_ = .220). This fixed-effects procedure has been used in multiple previous studies and is argued to be a more robust approach for computational fMRI (41, 42, 43).

At the second level, GLMs including group membership alongside covariates of age, sex, handedness, adjusted caudate volumes, and depression scores were estimated for model-free and model-based contrasts specified at the first level. The results of these second-level GLMs were imported into the MarsBaR for ROI analysis in which mean GLM parameter (β) estimates within regions of interest were used to either confirm replication of previous results or test our hypotheses.

### ROI Approach for Replication Analysis

To confirm replication of previously reported results using this task, we assessed the significance of parameter estimates within spherical ROIs defined from previously reported results. We created 6-mm spherical masks seeded at [ ±12, 10, −10] for the left and right ventral striatum, [±30, 28, −6] for the bilateral insula, and [−1, 27, −18] for the medial prefrontal cortex (29,44). Based on previous work, we assessed activity in these regions for the contrasts gain cue > neutral cue, loss cue > neutral cue, win money > lose money, lose money > win money. We also assessed activity in these regions for *Q* values and RPE. No exploratory whole-brain group differences were assessed.

### ROI for Reward Bias Analysis

To restrict our search for group differences to relevant brain regions, we first sought to determine regions that showed a reward bias in our task. Unbiased ROIs were derived from shared activity across both groups using the contrasts winning money > losing money or gain-predicting cues > loss-predicting cues after whole-brain voxelwise correction at familywise error *p* < .05 with a minimum cluster extent of 10. These voxels showed increased activity for rewards or reward-predicting cues compared with that for losses or loss-predicting cues. Clusters in the left and right striatum and prefrontal cortex were identified. These clusters were extracted using the MarsBAR toolbox in SPM. Striatal clusters were bounded by Automated Anatomical Labelling Atlas masks for the left and right caudate (see Figure S3). Previous work both in patients with HD and with this task suggests lateralization of the fMRI signal, and so left and right striatal clusters were kept separate (24,29).

Second-level GLMs were imported into MarsBAR, and average parameter (β) values within each ROI were estimated by subject. To test our hypothesis, gain cue > loss cue and the win money > lose money contrasts were compared between groups, using the contrast HDGCs > control subjects, correcting for age, sex, handedness, adjusted caudate volume, and depression scores. For each of these contrasts, differences were assessed in 3 ROIs (left and right ventral striatum and medial prefrontal cortex). As such, we tested for a difference between our groups using 2 contrasts, one at cue presentation and the other at outcome presentation, across 3 ROIs. To correct for this multiple comparison across these 6 texts, a stringent Bonferroni threshold of *p* < .008 was considered significant. Confirmatory analysis was then performed using only the appropriate 6-mm spherical ROIs defined from the literature as described above.

Model-based fMRI analysis was then used to assess whether neural correlates of *Q* value or RPE error differed by group. As above, analyses were performed in MarsBAR using the contrast HDGCs > control subjects for each of the 3 ROIs, with a threshold of *p* < .008 considered significant.

### Data Availability

Data will be shared on reasonable request post publication in accordance with Wellcome Trust open access data-sharing policy.

## Results

We recruited 35 HDGCs and 35 closely matched HD gene-negative control subjects. There were no differences in National Adult Reading Test scores, current depressive symptoms (Table 1 and Figure S4), or core cognitive tests typically sensitive to HD: Stroop word (*t* = −1.33, *p* = .19) and Stroop color reading (*t* = −1.06, *p* = .29), Symbol Digit Modalities Test (*t* = −0.43, *p* = .67), and verbal fluency (*t* = −1.58, *p* = .12). No significant caudate atrophy, typically a highly sensitive marker of HD (15), was seen in the HDGC group (Table 1 and Figure S5). The HDGCs had an average CAG length of 41.8 (±5.7), giving a mean estimated years to onset of 26.1 years (±5.5 years) based on the established Langbehn formula (5). The median unified HD motor score in this group was 0, with a maximum of 5, indicating that this group was definitely premanifest.

### Task Performance

**Figure 1:**
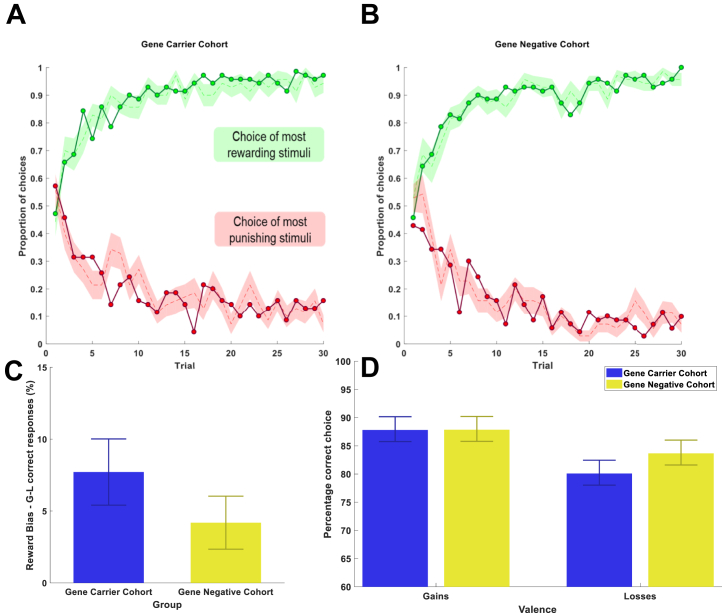
Both groups performed the task well, with no group differences. Participants in the **(A)** HDGC (*n* = 35) and **(B)** control (*n* = 35) groups learned to choose the most rewarding symbol in the gain (G) trials and avoid the most punishing symbol in the loss (L) trials. Mean participant choice data are shown by the dashed lines, with shaded regions showing ±SEM, green for gains and red for losses. The connected dotted green and red lines show mean computational model performance for gains and losses, respectively, showing computational model–predicted behavior well in both groups. **(C)** Both groups showed marginally better performance in learning from gains than in learning from losses with small positive reward bias (mean percentage correct in gains minus loss). **(D)** Percentage correct in gains and losses by group. Bars show mean correct, with error bar showing ±SEM. No significant group difference was observed in these metrics.

Participants completed an RL task in which they had to learn to choose between pairs of abstract stimuli associated with rewarding and punishing outcomes, as shown in Figure S1 (23,29). Both groups learned to choose the most rewarding symbol and avoid the most punishing symbol (Figure 1A, B). Both groups also showed more correct responses for gains than losses (gene carriers: *t* = 3.35, *p* = .002; gene negative: *t* = 2.27, *p* = .03). However, this behavioral reward bias was not significantly different between the groups (*t* = 1.19, *p* = .28) (Figure 1C). There was no significant difference in the percentage correct in either gains or losses between groups (*z*_gains_ = −0.11, *p*_gains_ = .91, *z*_losses_ = −.73, *p*_losses_ = .46) (Figure 1D).

### Computational Modeling of Behavior

**Figure 2:**
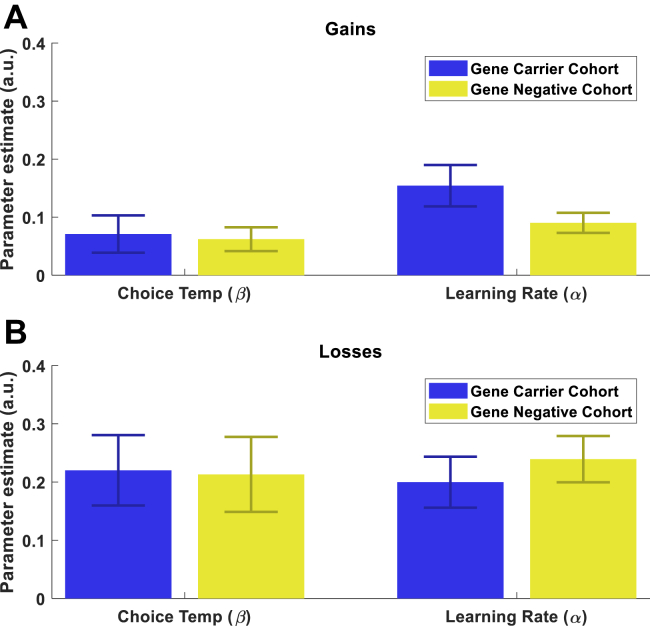
Computational model parameter estimates by group and valence. Mean parameter estimates for *Q*-learning model (±SEM) for each group in **(A)** gains and **(B)** losses—no significant group difference was observed. a.u., arbitrary units.

A *Q*-learning model fit behavior well in both groups (Figure 1A, B). Mean fitted groupwise parameter estimates for the learning rate and choice temperature from this model are shown in Figure 2. There was no significant parameter difference between the groups in either gains or losses (α_gain_: *z* = −1.22, *p* = .22; β_gain_: *z* = −0.41, *p* = .68; α_loss_: *z* = −1.29, *p* = .19; β_loss_: *z* = −0.47, *p* = .20).

### Robust Activation of Frontostriatal Regions Associated With Task

**Figure 3:**
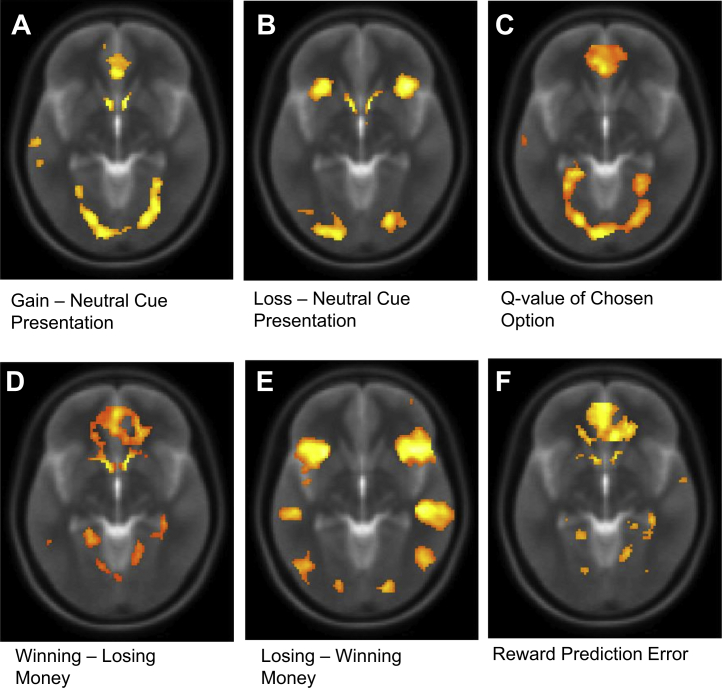
Neural activation in response to **(A–C)** choice and **(D–F)** outcome in task, for illustration purposes only. Neural activation **(A–C)** at cue presentation and **(D–F)** at outcome presentation across all subjects shown in the axial plane at an uncorrected threshold *p* < .001 with a cluster extent of 10 contiguous voxel.

As shown in Tables 2 and 3, we replicated, using spherical ROIs defined from existing literature, canonical neural findings associated with performance in an RL task. Bilateral ventral striatum (VS) activity was seen in response to gain and loss cues compared with neutral cues, reflecting learned stimulus value and associated with the RPE. Medial prefrontal cortex (mPFC) activity correlated with positive stimulus valence, the *Q*-value regressor, and RPE. Bilateral insula activation was associated with loss cue onset and the inverse, or punishment, prediction error. Figure 3 (and Figure S6) shows task activity at the whole-brain level across both groups at a liberal threshold, for illustration purposes only.

**Table 2:** Replication Analysis at Cue Presentation Across HD Gene–Positive and HD Gene–Negative Groups

	Gain > Neutral Cue		Loss > Neutral Cue		*Q* Value	
	*t*	*p*	*t*	*p*	*t*	*p*
Left VS	3.61	<.001	4.76	<.001	−0.018	.5
Right VS	5.77	<.001	6.77	<.001	−0.014	.51
Medial PFC	2.75	<.004	−1.71	.95	5.61	<.001
Bilateral Insula	1.22	.11	6.33	<.001	Not tested	Not tested

*N* = 70.

The table describes the 1-sided *t* statistic and corresponding *p* value whether activity in regions of interest, defined from existing literature, was positively associated with contrast at cue presentation.

HD, Huntington's disease; PFC, prefrontal cortex; VS, ventral striatum.

**Table 3:** Replication Analysis at Outcome Presentation Across HD Gene–Positive and HD Gene–Negative Groups

	Win > Lose Money		Lose > Win Money		Reward Prediction Error	
	*t*	*p*	*t*	*p*	*t*	*p*
Left VS	5.67	<.001	Not tested	Not tested	3.96	.001
Right VS	6.19	<.001	Not tested	Not tested	3.37	.006
Medial PFC	5.52	<.001	Not tested	Not tested	5.54	<.001
Bilateral Insula	Not tested	Not tested	5.47	<.001	−6.02	<.001^a^

*N* = 70.

The table describes the 1-sided *t* statistic and corresponding *p* value testing whether activity in regions of interest, defined from existing literature, was positively associated with contrast at outcome presentation.

HD, Huntington's disease; PFC, prefrontal cortex; VS, ventral striatum.

a Insula activity was positively associated with the punishment prediction error.

### Evidence of Ventral Striatal Reward Bias at Cue Onset in HDGCs

We identified clusters in the left and right VS and mPFC, stringently defined and unbiased with respect to group, that showed a reward bias in our task (see Methods and Materials) (ROIs are shown in Figure S3).

After adjusting for covariates of age, sex, handedness, adjusted caudate volume, and depression scores, we assessed whether mean parameter estimates in these regions were greater in HDGCs than in control subjects using 2 reward bias contrasts. Firstly, at cue presentation we compared gain cue presentation with loss cue presentation. Likewise, at outcome presentation we compared activity for winning and losing money. More positive estimates in these contrasts would suggest an asymmetry between gain and loss learning. To account for testing these 2 contrasts in our 3 ROIs, we used a stringent Bonferroni threshold of *p* < .008.

**Figure 4:**
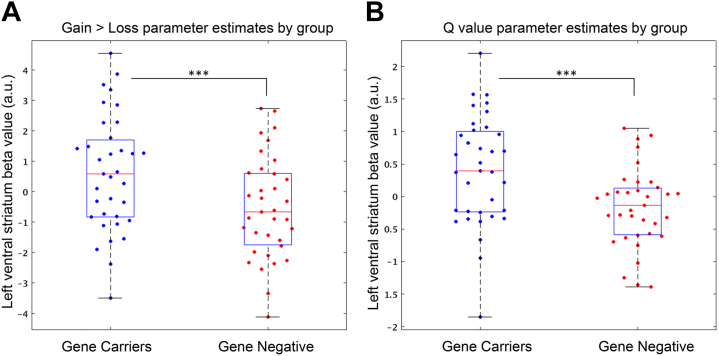
Left ventral striatum β values for reward bias contrasts, by group. Individual left ventral striatum parameter estimates by subject in each group for **(A)** gain cue minus loss cue activity and **(B)***Q*-value regressor. ∗∗∗Group difference, adjusting for effects of age, sex, and handedness, adjusted caudate volume, and depression scores, with significance of *p* < .005. a.u., arbitrary units.

At cue presentation, we found that the HDGC group, compared with the control subjects, showed an enhanced left VS response comparing gain cue presentation with loss cue presentations (*t* = 2.98, *p*_uncorrected_ = .002). This effect was not seen in the right VS (*t* = 0.69, *p*_uncorrected_ = .25) or the mPFC (*t* = 0.68, *p*_uncorrected_ = .25). Individual subject parameter estimates by group are shown in Figure 4A. To ensure that these results were not influenced by the choice of ROI, a 6-mm spherical ROI was created in the left VS at the coordinates described by Pessiglione *et al.* (29), and results showed the same difference between groups (*t* = 2.36, *p* = .01).

No significant difference between groups was seen at outcome presentation comparing winning money to losing money in any of the 3 ROIs (left VS: *t* = 1.07, *p* = .14; right VS: *t* = 1.03, *p* = .16; mPFC: *t* = 0.40, *p* = .35).

### Ventral Striatal Reward Bias Seen in Striatal Response to Model-Derived Value Estimates

In a complementary analysis, we compared activity in our 3 ROIs using regressors extracted from the computational model, the *Q* value, and the RPE (δ). Here, we use these regressors as complementary probes to the model-free fMRI analysis. Both regressors are positive in gain conditions and negative in loss conditions. More positive parameter estimates reflect a wider difference in neural activity between gain and loss domains in the cue and outcome phases of the task, for *Q* value and RPE, respectively. As above, we hypothesized that this would occur in the HDGC group compared with control subjects, and as before, we considered a Bonferroni threshold of *p* < .008 to be significant. As before, we adjusted this analysis for covariates of age, sex, handedness, adjusted caudate volume, and depression scores.

In keeping with the model-free analysis, we found that the HDGC cohort compared with the control subjects had higher parameter estimates associated with the *Q*-value regressor in the left VS (*t* = 3.56, *p*_uncorrected_ = .0004) but not the right VS (*t* = 0.82, *p*_uncorrected_ = .21) or the medial prefrontal cortex (*t* = 1.14, *p*_uncorrected_ = .13). Using the independent spherical left VS ROI, we found the same differences between groups (*t* = 2.69, *p* = .005). Individual parameter estimates by group are shown in Figure 4B. We found no evidence of a difference in the β values associated with RPE-related activity in the HDGC cohort (left VS: *t* = −0.1, *p* = .54; right VS: *t* = −0.34, *p* = .63; mPFC: *t* = −0.46, *p* = .68).

Neither the parameter estimates in the gain versus loss contrast (*r* = −.03, *p* = .89) nor the *Q*-value parameter estimates (*r* = −.11, *p* = .53) in the HDGC group correlated with the disease burden score.

### More Robust Response to Loss Cues in Control Subjects

To display the reward bias described above, either HDGCs should show a greater response to gain cues than control subjects or the control subjects should display greater response to loss cues than HDGCs, or both. Post hoc, using the unbiased task-defined left VS ROI, we tested these hypotheses by comparing valence cue onsets with neutral cue onsets. HDGCs did not show an enhanced striatal response comparing gain cues with neutral cues (*t* = 0.95, *p*_uncorrected_ = .17); however, control subjects did show significantly increased response to loss cues than neutral cues (*t* = 2.03, *p*_uncorrected_ = .02).

## Discussion

In this study, we recruited a cohort of healthy HDGCs estimated to be 25 years from onset of motor symptoms, based on age and CAG length, and found that neural activity in response to stimulus valence and value was significantly different from matched control subjects in the striatum. No group difference was seen in behavior, cognitive scores, or caudate volumes. Taken together, these findings suggest that changes in the frontostriatal value networks may occur very early in the life of HDGCs; however, these changes are not sufficient to manifest as behavioral differences, 25 years before motor onset. To our knowledge, our findings are the earliest reported functional imaging differences between HDGCs and control subjects.

In a behavioral study, Palminteri *et al.* (23) reported an asymmetry between gain and loss learning, a reward bias, in HDGCs approximately 10 years from motor onset. Although they did not collect fMRI data, they found that these behavioral differences may be driven by computational differences at the choice phase, with HDGCs making noisier decisions in the loss frame. These findings are wholly in keeping with our fMRI findings. We also found a difference between our groups at the choice phase with some evidence that this difference is mediated by impaired loss cue representation in the VS. We found no difference at outcome or in our behavioral analysis. As both studies used the same task, the neural changes identified here may be antecedent to the behavioral and computational changes identified by Palminteri *et al.* (23) and may be predicted to occur in our cohort in approximately 15 years. These findings also raise three further possibilities. Firstly, they suggest that a window exists in which functional neural changes occur in HDGCs, before measurable atrophy and corresponding behavioral changes. Secondly, these findings may be in keeping with early dysfunction of the indirect pathway in HDGCs, which is lost at a greater rate in HD and thought to contribute to loss learning and avoidance (14,31,33,35, 36, 37). Finally, this study adds to the growing body of evidence that suggests that limbic and reward processing may be among the most sensitive and early cognitive markers of HD (8,23,24,45,46).

It has been hypothesized, secondary to disinhibition resulting from the loss of striatal medium spiny neurons, that dopaminergic signaling in HD follows an inverted U-shape in which low levels of dopamine are found later in HD whereas increased dopamine signaling is found earlier in the disease (47). In keeping with this model, using a reversal learning task, Nickchen *et al.* (22) reported the loss of RPE signaling in the left VS of patients with manifest HD, especially those more severely affected. Pharmacological induction of a hyperdopaminergic state using our task has been shown to enhance RPE signaling; however, we found no difference between our HDGC group and control subjects in striatal RPE activity to suggest exaggerated dopamine signaling (29,48). It may be that participants in our HDGC group were too far from onset for dopamine dysregulation to manifest as measurable differences in blood oxygen level–dependent activity. Similar to our results, in a nonlearning task with gain and loss domains, Enzi *et al.* (24) reported that left ventral striatal activity in HDGCs 10 years from onset showed impaired striatal representation of punishment cues in the left VS. Unlike our study, they did not find differences between HDGCs far from onset and control subjects. Aside from differences in sample size, these findings suggest that learning tasks may be more sensitive in very premanifest HD.

We believe that our study has a number of strengths. We successfully recruited a unique cohort of HDGCs estimated to be more than 2 decades from motor onset and a well-matched cohort of control participants. We were able to demonstrate that our groups were not significantly different in tests traditionally sensitive to HD. We used a task with existing data in patients with HD and gene carriers closer to onset to allow us to better interpret our results, and we were able to replicate canonical results from the literature. Given both prior literature and our hypotheses, we restricted group comparison to a limited number of regions. ROIs were defined using task data to identify voxels that showed hypothesis-relevant activity. Although task derived, a stringent statistical threshold was used to identify these clusters to limit the inclusion of false-positive voxels. These regions were identified based on shared activity across both groups and so were not biased to group differences. We also then ran a confirmatory analysis with a spherical ROI at independently reported coordinates and found the same group differences.

We also acknowledge that our study has the following limitations that readers may wish to consider. Our study design was cross-sectional and not powered to determine whether the effect that we found progresses as participants grow closer to disease onset. Although we lack longitudinal data, existing published HD data using this task may serve as an indicator of how task performance may change as the disease progresses. We also do not claim that our findings clearly represent disease per se; however, in conjunction with published data from the same task, we believe that our findings are likely to be antecedent of reward processing deficits that may emerge later. Recent work has shown the disease-related change particularly in fluid biomarkers can also be found at this point (38). Unlike previous work, we found differences only in fMRI data and not in behavioral data. Firstly, given that effect sizes for behavioral differences on cognitive tasks diminish in gene carriers further from disease onset, gross behavioral differences in our HDGCs may have been surprising (11). It is important to highlight, however, that lack of behavioral differences in this paradigm may also be related to task difficulty. Behavioral difference in reward learning may be seen this far from clinical onset with more challenging learning tasks.

### Conclusions

Here, we demonstrate that healthy HDGCs approximately 25 years from motor onset show an exaggerated striatal response to gain-predicting cues compared with loss-predicting cues in a computational fMRI study. This difference between gene carriers and matched control subjects was also seen in striatal activity related to the predicted value of choice derived from a computational model. These results suggest that aberrant neural coding of valence and value may be one of the earliest features of carrying the HD gene, occurring decades before onset. These changes are not accompanied with robust behavioral changes, suggesting that an important window exists—where neural changes occur in HDGCs but before these changes drive potentially hard to recover behavioral impairment.
