# Supplementary material for: Aberrant Striatal Value Representation in Huntington's Disease Gene Carriers 25 Years Before Onset
Source: Biol Psychiatry Cogn Neurosci Neuroimaging. 2021 Sep;6(9):910–8. doi: 10.1016/j.bpsc.2020.12.015 (PMC8423628; doi:10.1016/j.bpsc.2020.12.015)
Supplement: Supplementary Material [file mmc1.pdf]

Aberrant Striatal Value Representation in Huntington's Disease Gene Carriers 25 Years Before Onset

Supplemental Information

Supplementary Methods

Subject details:

Both groups underwent neurological examination by experienced HD clinicians (A.N. and P.Z.) to confirm the absence of gross motor signs associated with HD. Participants underwent a cognitive assessment including the National Adult Reading Test as a measure of premorbid intelligence and core cognitive measures sensitive to HD - the Symbol Digit Modality Test (SDMT) (total correct in 90s), Stroop word and colour reading (total correct in 45s) and verbal category fluency (scored as unique responses in 60s). There was no group difference in self-reported prior diagnoses of depression or current SSRI use (see Fig. S4).

One participant was diagnosed with a coincidental serious neurological condition based on neuroimaging and excluded. Two participants could not complete fMRI scanning due to claustrophobia. Data from these three participants were not analysed and participants were excluded and replaced.

Task description:

Symbols were placed above and below a central fixation cross. To choose the upper stimulus participants were required to make a 'Go' response and press a button on a fMRI compatible button box using their right hand. To choose the lower symbol participants withheld response for 3 seconds. Symbol position was random in each trial, so position and value were orthogonal. After either a button press or 3 second delay period, choice was displayed for a jittered interval ranging from 0.5–2 seconds drawn from an exponential distribution. Outcome was then displayed for 3 seconds followed by a jittered inter-trial interval (ITI) ranging from 2-6 seconds drawn from an exponential distribution.

Computational modelling fitting details:

Parameters were fit to maximize the likelihood of the subject's choice using the *fmincon* function (alpha bounded between 0 and 1, inverse beta bounded between 0 and 100). The search was initiated at multiple, 40, random start points in parameter space to avoid local minima. Goodness of model fit is

reported with McFadden's pseudo-R^2 calculated as 1 – log likelihood of the model divided by log likelihood of null model (in which choices are determined by chance). This model reinforcement learning model provided good model fits for both gains and losses (McFadden's pseudo-R^2_{gain} = 0.65 +/- 0.27, pseudo-R^2_{loss} = 0.48 +/- 0.25, mean ± STD) with no difference in fits between the groups for either gains or losses (Z_{gains} = 0.81, p = 0.42, Z_{losses} = -0.83, p = 0.40). Furthermore, comparing this model to a three-parameter model (including a reward multiplier term) and models in which the initial q value (q0) was both 0 and treated as a free parameter, the model described above performed best in terms of model comparison based on summed BICs across participants and valences (Fig. S2) Go and No-Go response in gains and loss conditions were approximately 50% as expected and not significantly different between groups and was not considered further (Gain-Go Controls: 47.2% ± 0.07, HDGC: 46.9% ± 0.07, p_{gains} = 0.86, Losses-Go: Controls: 45% ± 0.08, HDGC: 48% ± 0.08 p_{losses} = 0.09, (mean ± STD)).

fMRI image acquisition:

Each volume contained 48 slices with a 3mm^3 resolution. Volume TR was 3.36 seconds with a slice tilt of -30 degrees, a Z-shim of -0.4 and ascending slice acquisition order. T1 weighted images were collected for structural alignment and volumetric analysis. The 3D T1-weighted sequence was an optimised MPRAGE protocol, with an echo time (TE) of 3.34ms and a repetition time (TR) of 2530ms. The inversion time was 1100ms, and the flip angle was 7 degrees. The field of view was 256x256x176mm, with 1mm isotropic voxels. Parallel imaging acceleration (GeneRalized Autocalibrating Partial Parallel Acquisition, GRAPPA, acceleration factor (R)=2) was applied along with 3D distortion correction and pre-scan normalisation. Following the task, field maps were acquired for unwarping. Physiological monitoring of heartbeat and breathing were recorded for 31 of 35 participants. Excluding participants with missing physiological monitoring did not influence results (Supplementary Table S1).

fMRI pre-processing:

Images were processed using SPM12. Images were un-warped using acquired field maps, slice-time correction to the middle slice, corrected for motion, and then warped into Montreal Neurological Institute (MNI) template space and spatially smoothed with a Gaussian kernel of 6-mm. Following unwarping and warping to standard space images underwent manual quality check. No subjects were excluded for excessive head motion or quality assurance reasons.

Structural imaging processing:

All T1-weighted scans passed visual quality control checks for the absence of significant motion or other artefacts before processing. Bias correction was performed using the N3 procedure. An automated segmentation procedure, Multi-Atlas Label Propagation with Expectation-Maximisation based refinement (MALP-EM), was used to measure caudate volume (1). All settings were applied using default parameters, except for the inclusion of a brain mask for each participant based on a previously generated whole-brain region derived from semi-automated delineation. MALP-EM has been validated for use in HD.(2) A validated semiautomated segmentation procedure performed via Medical Image Display Analysis Software (MIDAS) was used to generate volumetric regions of total intracranial volume (TIV) (3,4). Caudate volume was adjusted for TIV using the formula:

$$Caudate_{adjusted} = \frac{TIV_{mean}}{TIV_{individual}} \times caudate_{individual}$$

Whereby:

$Caudate_{adjusted}$= adjusted caudate volume for the participant

TIV_{mean} = mean TIV for whole cohort

$TIV_{individual}$ = TIV for the participant

$Caudate_{individual}$= raw caudate volume for the participant

All scans and regions underwent visual quality control by experienced raters (R.S. and E.J.) to ensure that there were no scan artefacts or errors in the delineation of brain regions. No scans or segmentations failed after visual quality control.

Supplementary Tables

	Gain > Loss Cues	Q-values	Win > Losing	RPE
**Left VS**	t = 2.76, p = 0.004	t = 3.26, p < 0.001	t = 1.20, p = 0.12	t = 0.22, p = 0.41
**Right VS**	t = 0.58, p = 0.28	t = 0.77, p = 0.22	t = 1.74, p = 0.04	t = 0.15, p = 0.44
**mPFC**	t = 0.99, p = 0.16	t = 1.40, p = 0.08	t = 0.85, p = 0.20	t = 0.01, p = 0.50

Supplementary Table S1: Reward bias results excluding participants with missing physiological data. Related to STAR Methods: Physiological data was missing for four participants due to technical difficulties (gene carriers = 3, controls = 1). Excluding these participants from the analysis did not influence the results as shown in the table above. This table reports the t-statistic and p-value of the difference in parameter estimates for each contrast used to test the 'reward bias' hypothesis. Bonferroni threshold of < 0.008 is considered significant. As described in the main text, gene carriers showed more positive parameter estimates in the gain > loss cue contrasts and the complementary Q-value contrast, adjusted for age, gender, handedness, depression scores and adj. caudate volume.

Supplementary Figures:

Task interface and design

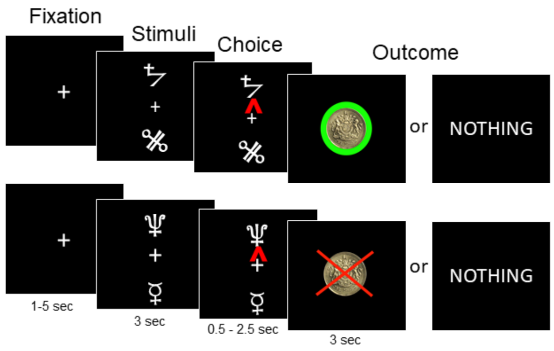

Supplementary Figure S1: Reinforcement learning task with gain and loss domains was performed by subjects.

Participants were presented initially with a fixation cross. This was replaced by two abstract symbols. Participants saw three such abstract pairs – a 'gain pair', 'loss pair' and 'neutral pair'. Using a button box in their right-hand participants were instructed to press the button to choose the top symbol or withhold their response for 3 seconds to choose the lower symbol. Their choice was displayed with a red marker before the outcome was revealed. In the gain pair, one symbol was associated with reward with a probability of 0.8 and the other with a probability of 0.2. Similarly, in the loss pair, one symbol was associated with losing reward with a probability of 0.8 and the other with a probability of 0.2. Reward in the gain condition was signified with a picture of £1 with a green halo and loss signified with red cross through the £1. Alternatively, participants saw the word 'Nothing'. In the neutral condition, participants saw an empty disc or the word 'Nothing'. Timings are for transition are shown in figure and described in Methods.

Model comparison with BIC

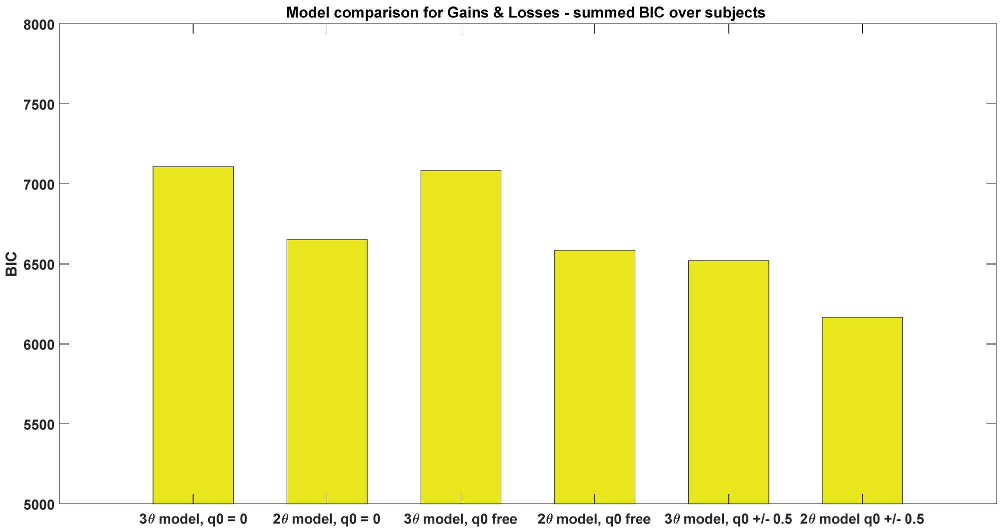

Supplementary Figure S2: Model comparison of competing computational models related to STAR Methods.

3 parameter model included a reward magnitude as described in Palminteri *et al* (2012). q0 = 0, q0 free and q0 +/- 0.5 describe models in which q0 for both stimuli was initialised as 0 in gains and loss conditions, q0 was treated as a free parameter and when q0 was initialised +0.5 in the gain frame and -0.5 in the loss frame. The 2-parameter model with q0 initialised at +0.5 in the gain frame and -0.5 was the winning model in the model comparison, scoring the lowest BIC score (2ϴ model, q0 +/- 0.5). This model was used in the main analysis. BIC: Bayesian Information Criterion.

Task-derived ROIs

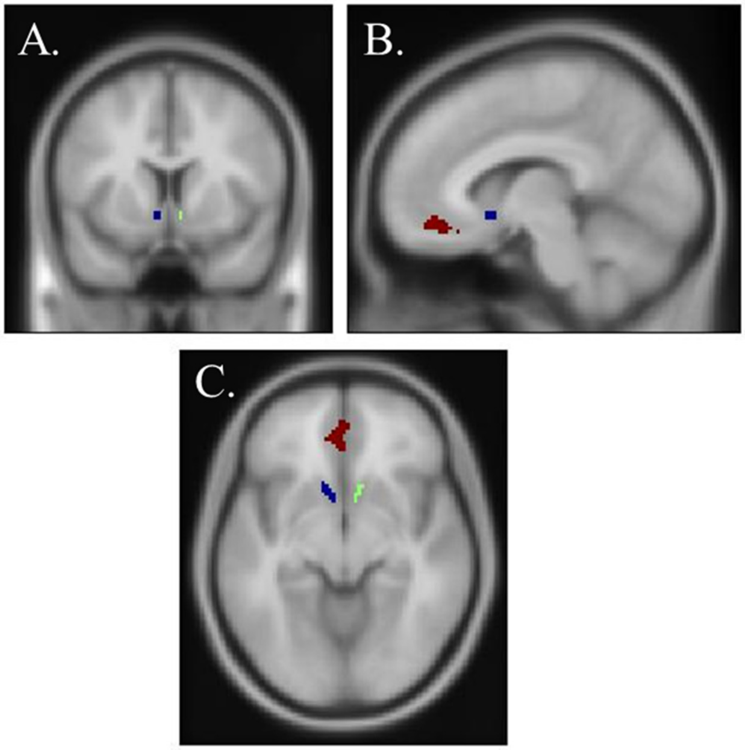

Supplementary Figure S3: Masks for reward response regions of interest used for reward bias analysis.

Masks used as task-derived regions of interest which showed reward responsiveness – greater activity in response to winning money as compared to losing money or gain versus loss cue presentation. Clusters were formed from voxels which survived FWE correction across the whole brain at a threshold of $p < 0.05$ across both groups ($n = 70$) in either contrasts. Striatal clusters were identified only in the Win > Lose money contrast. Blue ROI is the left ventral striatum; green ROI is the right ventral striatum and red ROI is the medical PFC superimposed on T1 image A. shows the coronal slice through the striatal ROIs, B. shows the sagittal slice showing left VS and medial PFC and C. shows the axial slice containing all three ROIs.

Distribution of current depressive scores

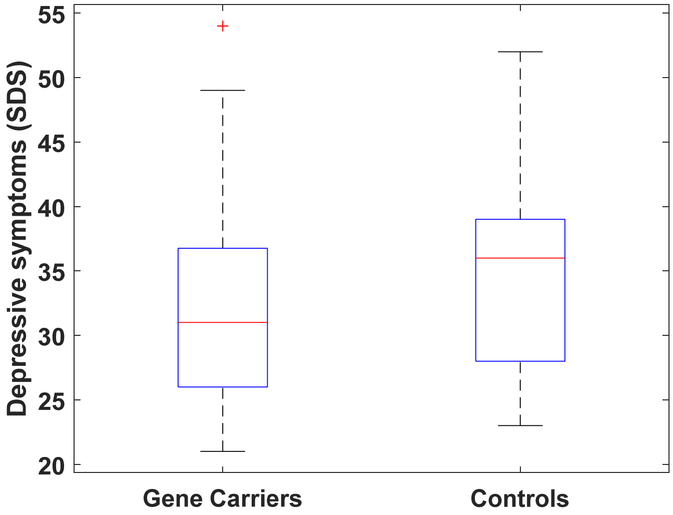

Supplementary Figure S4: Depressive scores by group, related to Table 1. Depressive scores as recorded by the Zung Depression Scale (SDS). HDGC: 32.2 (+/-8.60), Controls: 34.6 (+/- 7.4) (means +/- std). No difference was seen between groups ($t = -1.2$, $p = 0.22$). Scores < 50 are considered to be in the normal range with scores 50-59 indicating possible mild depression. Groups also did not differ on previous self-reported diagnoses of depression (GC: 7/35, HC: 5/35, $\chi = 0.4$, $p = 0.53$) or current SSRI use (GC: 4/35, HC: 2/35, $\chi = 0.73$, $p = 0.39$)

Adjusted caudate volumes by group (adjusted for TIV)

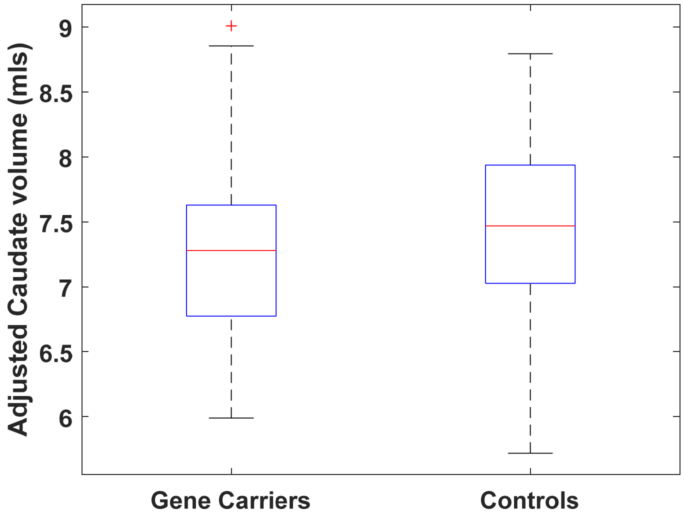

Supplementary Figure S5: Adjusted caudate volume by group, related to Table 1. Caudate volumes adjusted by total intracranial volume (see methods) showed no significant atrophy between groups ($t = -0.84$, $p = 0.40$)

Task activation

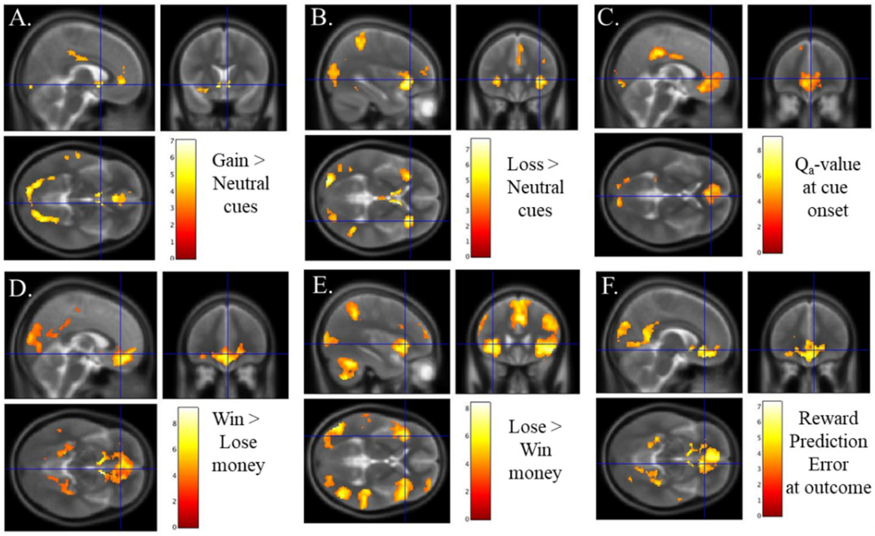

Supplementary Figure S6: Neural activation during choice and outcome phase in task in three planes related to Figure 4. Task contrasts described in main text shown in three planes at a liberal threshold (p < 0.001 uncorrected with cluster forming extent (k) of 10. A-C showing contrasts at cue presentation (A. Gain > Neutral, B. Loss > Neutral, C. Q-value of chosen option). D-F showing contrasts at outcome presentation (D. Win > Lose money, E. Lose > Win money, F. Reward prediction error)

Supplemental References

1. Ledig C, Heckemann RA, Hammers A, Lopez JC, Newcombe VFJ, Makropoulos A, *et al.* (2015): Robust whole-brain segmentation: Application to traumatic brain injury. *Med Image Anal* 21: 40–58.
2. Johnson EB, Gregory S, Johnson HJ, Durr A, Leavitt BR, Roos RA, *et al.* (2017): Recommendations for the Use of Automated Gray Matter Segmentation Tools: Evidence from Huntington's Disease. *Front Neurol* 8: 519.
3. Freeborough PA, Fox NC, Kitney RI (1997): Interactive algorithms for the segmentation and quantitation of 3-D MRI brain scans. *Comput Methods Programs Biomed* 53: 15–25.
4. Malone IB, Leung KK, Clegg S, Barnes J, Whitwell JL, Ashburner J, *et al.* (2015): Accurate automatic estimation of total intracranial volume: a nuisance variable with less nuisance. *Neuroimage* 104: 366–72.
